# Editorial: Genetics, Genomics and –Omics of Thermophiles

**DOI:** 10.3389/fmicb.2017.00560

**Published:** 2017-04-03

**Authors:** Kian Mau Goh, Kok-Gan Chan, Rajesh Kumar Sani, Edgardo Rubén Donati, Anna-Louise Reysenbach

**Affiliations:** ^1^Faculty of Biosciences and Medical Engineering, Universiti Teknologi MalaysiaSkudai, Malaysia; ^2^Division of Genetics and Molecular Biology, Institute of Biological Sciences, Faculty of Science, University of MalayaKuala Lumpur, Malaysia; ^3^Department of Chemical and Biological Engineering, South Dakota School of Mines and TechnologyRapid City, SD, USA; ^4^CINDEFI (CCT, La Plata-CONICET, UNLP), Facultad de Ciencias Exactas, Universidad Nacional de La PlataLa Plata, Argentina; ^5^Department of Biology, Portland State UniversityPortland, OR, USA

**Keywords:** comparative genomics, extremophile, hot spring, hyperthermophile, thermozyme, metagenome

Thermophilic Archaea and Bacteria occupy heated environments. Advancement of next-generation sequencing (NGS), single-cell analyses, and combinations of –omics and microscopic technologies have resulted in the discovery of new thermophiles. This e-book consists of a review, and 10 original articles authored by 94 authors. The main aim of this Research Topic of Frontiers in Microbiology was to provide a platform for researchers to describe recent findings on the ecology of thermophiles using NGS, functional genomics, comparative genomics, gene evolution, and extremozyme discovery.

The review by DeCastro et al. discussed the approaches currently available in assessing the taxonomy and functional metagenomics of thermophiles in high temperature environments. The review also provides limitations or challenges for each approach in the discovery of novel thermozymes that include lipolytic enzymes, glycosidases, proteases, and oxidoreductases.

Nearly 50 years ago, Thomas Brock was among the earliest researchers who elucidated the existence of living organisms in hot springs in Yellowstone National Park, YNP (Brock, 1967). In this e-book, Thiel et al. revisited Mushroom Spring (60°C) and examined the microbial diversity in the orange-colored undermat using NGS shotgun sequencing and 16S rRNA amplicon analyses. The phylum *Chloroflexi* dominated 49% of total OTUs, followed by *Thermotogae, Armatimonadetes* (previously known as candidate division OP10), *Aquificae, Cyanobacteria, Atribacteria* (candidate phylum OP-9/JS1), *Nitrospirae* and others. Thiel et al. showed that the dominant taxon, *Roseoflexus*, had high microdiversity of the 16S rRNA gene sequences which most likely represent different ecotypes with specific ecological adaptations. In a separate article, Saxena et al. performed shotgun metagenomic and 16S rRNA amplicons sequencing from samples collected from three Indian hot springs (43.5–98°C, pH 7.5–7.8). The alpha- and beta-diversity of thermophiles in seven distinct sites were compared and the authors concluded that the temperatures significantly affected the microbial community structure. These sites were dominated by phyla *Proteobacteria, Thermi, Chloroflexi, Bacteroidetes, Firmicutes*, and *Thermotogae*. Data from shotgun metagenome sequencing were used to assess hydrocarbon degradation pathways in the Anhoni hot spring. One of the interesting insights from Saxena et al. is that all enzymes involved in a particular hydrocarbon degradation pathway were not found in a single microbial species; therefore, the degradation could only be completed by consortium members of the microbial community.

Genomes of several hyper- and thermophilic bacteria were sequenced and reported in this e-book. *Dictyoglomus turgidum* has an optimum growth temperature (OGT) of 72°C. Brumm et al. analyzed genome content of *D. turgidum* from multiple aspects including metabolic pathways, polysaccharides degradation and transport, energy generation, DNA repair and recombination, and stress responses. *D. turgidum* has an abundance of glycosyl hydrolases, 16 of which were examined for their activities. *D. turgidum* can utilize most plant-based polysaccharides, except crystalline cellulose. Zarafeta et al. isolated a *Dictyoglomus* sp. Ch5.6.S from an *in situ* enrichment culture containing xanthan gum established in a Yunnan hot spring. A new hyperthermostable esterolytic enzyme (EstDZ3) was identified from the genome sequence. The EstDZ3 is likely a carboxylesterase as it reacts best on fatty acid esters with short to medium chain lengths. The enzyme exhibited a half-life of more than 24 h when incubated at 80°C. Clearly, both articles suggested *Dictyoglomus* is an interesting genus with biotechnological potential.

Three articles in this Research Topic provide novel insights into sulfur-metabolizing prokaryotes. Zhang et al. studied the evolution of six *Acidithiobacillus caldus* strains using comparative genomic approaches. The authors identified many mobile genetic elements and showed that gene gains and losses all drive the genomic diversification in this species. Dai et al. compared the genome of *Sulfolobus* sp. A20 with the *Sulfolobus solfataricus, Sulfolobus acidocaldarius, Sulfolobus islandicus*, and *Sulfolobus tokodaii*, and identified 1,801 core genes sequences. Genes in central carbon metabolism and ammonium assimilation are highly conserved in *Sulfolobus*. The genes for sulfur oxygenase/reductase and inorganic nitrogen utilization which are less conserved probably due to the presence or remnants of insertion sequence elements. The anaerobic *Thermosulfurimonas dismutans* S95^T^ was isolated from a deep-sea hydrothermal vent by Mardanov et al. and they showed that *T. dismutans* had the sulfur-disproportionating capability even without the need of direct contact of the cells to solid elemental sulfur. It is therefore likely that the soluble glutathione persulfide is the actual substrate entering the disproportionation pathway. Mardanov et al. proposed a model of sulfur metabolism and related pathways in *T. dismutans*.

Goh et al. reported the isolation and genome description of a new bacterium strain RA (OGT: 50–60°C) in the *Rhodothermaceae*. The strain RA is likely to be a new genus due to its low 16S rRNA and housekeeping genes (e.g., *recA, rpoD*, and *gyrB*) similarity to other genera in *Rhodothermaceae*. Goh et al. also compared the genome of this bacterium with *Rhodothermus marinus* DSM 4252^T^ and *Salinibacter ruber* DSM^T^ and showed that it has putative genes for adaptation to osmotic stress and survival in a high sulfidic hot springs. Using phylogenetic analyses and sequence similarity networks, Cardenas et al. reported the phylogenomic analysis of 2,631 representative rubrerythrins, a group of proteins involved in oxidative stress defense. The authors proposed that “aerobic-type” rubrerythrins underwent a separate adaptation process than that of the “cyanobacterial group.” Lastly, Irla et al. compared four different plasmids that were able to replicate in *Bacillus methanolicus*. They reported the effects of copy number, expression levels and stability of these plasmids in *B. methanolicus*. The article provided new tools for genetic engineering of *B. methanolicus*. We hope that this e-book can stimulate the research community to integrate –omics and bioinformatics tools in understanding the biology of heated environments and thermophiles.

## Author contributions

All authors listed, have made substantial, direct and intellectual contribution to the work, and approved it for publication.

### Conflict of interest statement

The authors declare that the research was conducted in the absence of any commercial or financial relationships that could be construed as a potential conflict of interest.
